# Conformational analysis and quantum descriptors of two new imidazole derivatives by experimental, DFT, AIM, molecular docking studies and adsorption activity on graphene

**DOI:** 10.1016/j.heliyon.2020.e05182

**Published:** 2020-10-06

**Authors:** Veena S. Kumar, Y. Sheena Mary, Kiran Pradhan, Dhiraj Brahman, Y. Shyma Mary, Goncagül Serdaroğlu, Ali Shokuhi Rad, M.S. Roxy

**Affiliations:** a Department of Physics, SN College, Kollam, Research Centre, University of Kerala, Kerala, India; b Department of Physics, Fatima Mata National College(Autonomous), Kollam, Kerala, India; c Department of Chemistry, St. Joseph's College, P.O. North Point, Dist. Darjeeling 734104, India; d Sivas Cumhuriyet University, Faculty of Education, Math. and Sci. Edu., 58140 Sivas TURKEY; e Department of Chemical Engineering, Qaemshahr Branch, Islamic Azad University, Qaemshahr, Iran

**Keywords:** Organic chemistry, Pharmaceutical chemistry, Theoretical chemistry, DFT, Imidazole, MEP, QTAIM, Docking

## Abstract

1-[2-(2-hydroxy-3-methoxy-5-(4-methoxyphenylazo)benzaldeneamino)ethyl]-3-methyl-3H-imidazole (HMY) and 1-[2-(2-hydroxy-3-methoxy-5-(4-methylphenylazo)benzaldene amino)ethyl]-3-methyl-3H-imidazole (HMM) were synthesized and characterized using spectral analysis. Conformational analysis has been achieved using potential energy scan for different rotable bonds for obtaining the lowest energy conformer. Conformer with minimum energy is obtained along the dihedral angle N30-C31-C34-N37. QTAIM analysis gives nature and strength of hydrogen bonding interactions. UV-Vis, electrostatic potential and chemical descriptors are analyzed. Interaction of HMY and HMM with graphene is analyzed in terms of SERS activity. Chemical reactivity descriptors were investigated for graphene-drug systems. NLO activity of parent drugs and its graphene complexes show good activity. The wavenumber downshift of different modes is noted. Title molecules exhibit inhibitory activity against cytochrome C peroxidase. Interactions with graphene sheets are theoretically predicted for the title compounds.

## 1 Introduction

Imidazoles are materials for chemical synthesis with remarkable biological activities [1]. Some derivatives are characterized by properties of electroluminescence and have flurophores in diodes that emit light [2, 3]. Azo compounds are industrial organic colorants and used in various fields, electronics, foods, drugs, cosmetics and textiles due to the versatile applications [4]. Imidazole drugs have uses in medical field of anticancer, antiviral, antibacterial and anti-diabetic activities [5, 6]. They are very important in materials chemistry as ionic liquids [7, 8] and in organic reactions as carbene precursors which are more stable [9]. Imidazole drugs show anti-hypertensive activities [10, 11]. They are also efficient corrosion inhibitors [12]. Khodja et al. recently reported a series of imidazole derivative's design, synthesis and biological evaluation [13]. Kandasamy et al. recently reported the synthesis of zinc binding groups based on imidazole inhibitors that target lung cancer [14]. Recently green synthesis of an ionic liquid is reported based on imidazole [15]. Shahi et al. presented synthesis and distribution of electron density in imidazole derivatives [16]. Mary et al. reported a number of imidazole derivative's spectroscopic studies [17, 18, 19, 20, 21, 22, 23]. In literature, broad research has been reported on graphene activity [24, 25, 26, 27, 28, 29]. Hydrophobic interaction is a major adsorption mechanisms of drugs with graphene [30, 31, 32, 33]. GQDs provide high SERS signals for detecting drugs due to electronic properties [34, 35, 36, 37, 38, 39, 40, 41, 42, 43]. Adsorption of drugs with graphene is reported by many authors experimentally and theoretically [44, 45, 46, 47, 48, 49, 50, 51, 52]. Coronene structures are reported as mimic of graphene [53, 54, 55]. Mary et al. reported the interaction of organic molecules with graphene/fullerene and doped graphene sheets [56, 57, 58, 59]. In the present study, DFT investigations, spectral analysis and docking study of title molecules, HMY and HMM was employed to determine the various properties including adsorption on coronene like graphene [60, 61, 62, 63].

## 2 Methods of calculation and experimental

**Figure 1:**
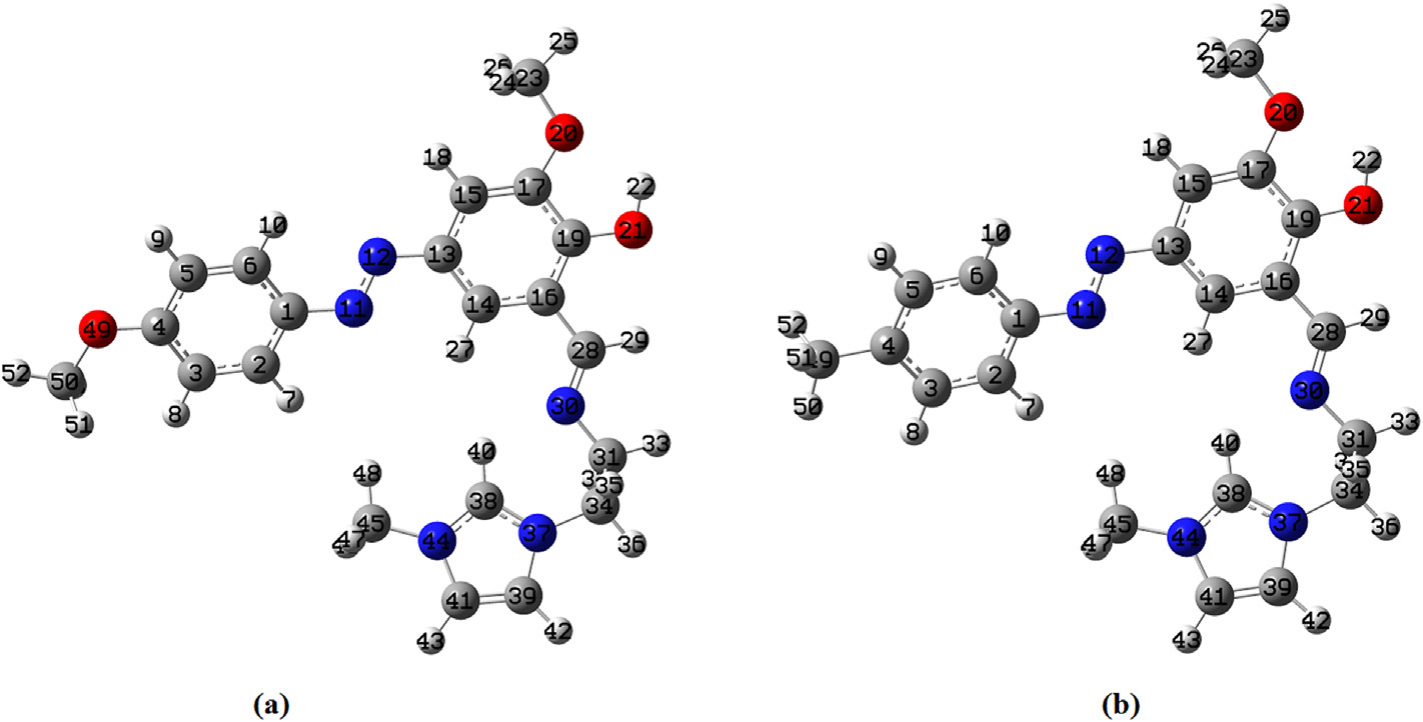
Optimized structure of (a) HMY and (b) HMM.

All calculations related to this study have been executed with computational chemistry software package Gaussian09 program [64]. In order to obtain correct structural feature of HMY and HMM (Figure 1), DFT theory with B3LYP with 6–311++G (d,p) basis have been taken. B3LYP is most popular DFT functional and widely used in density functional calculations [65, 66, 67, 68]. GAR2PED and Gasuview programs has been utilized to compute percentage potential energy distribution and hence to correctly assign vibrational wavenumbers of the title compound [69, 70]. QTAIM is a powerful method used to investigate the nature of all bonds in the course of HMY and HMM compounds [71, 72]. The QTAIM approach is useful to obtain electron density values and bonding characteristics of the configurations [71]. The charge density (ñ(r)), Laplacian of charge density (∇^2^(r)), ellipticities (å) are calculated by AIMALL program [72] with all default options, were used to evaluate nature of the interaction. Based on the QTAIM approach, every two interacting atoms were connected by the bond path (BP), and one point (saddle point) in the BP had a maximum value of electron density named bond critical point (BCP).

**Figure 2:**
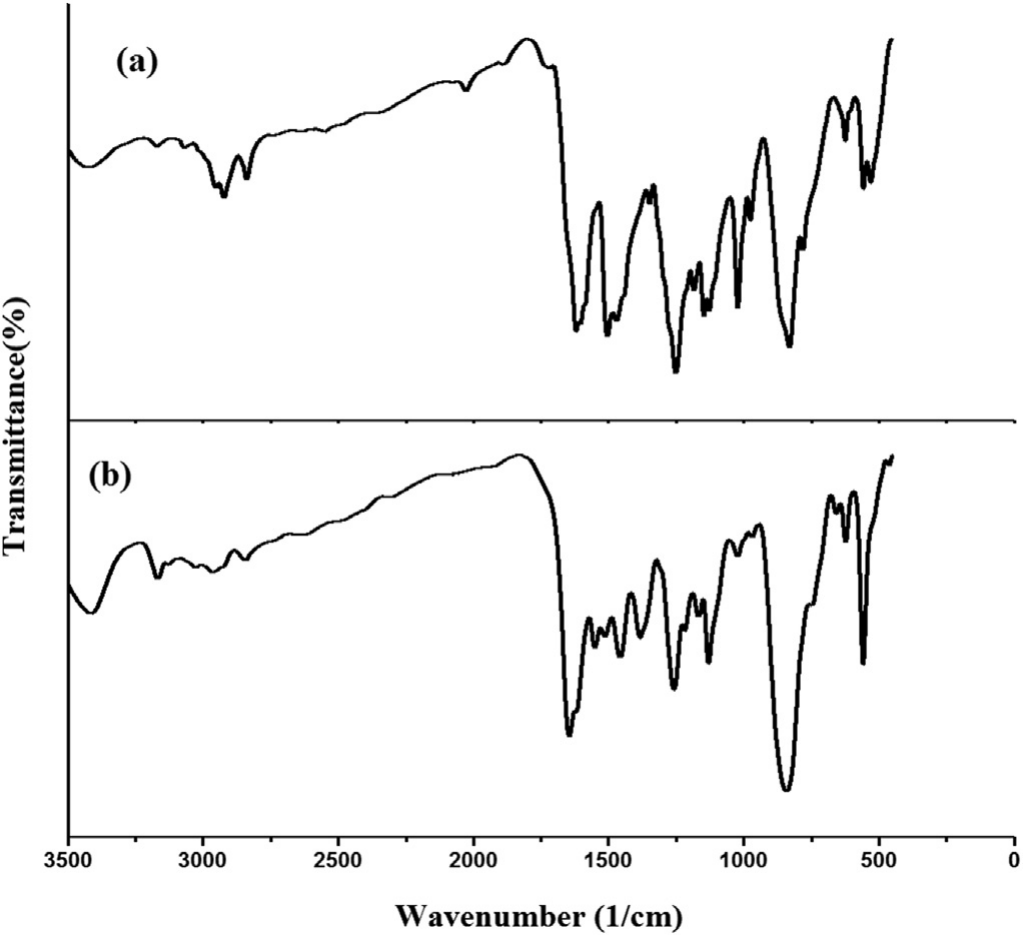
Experimental FT-IR curves of (a) HMY and (b) HMM.

**Figure 3:**
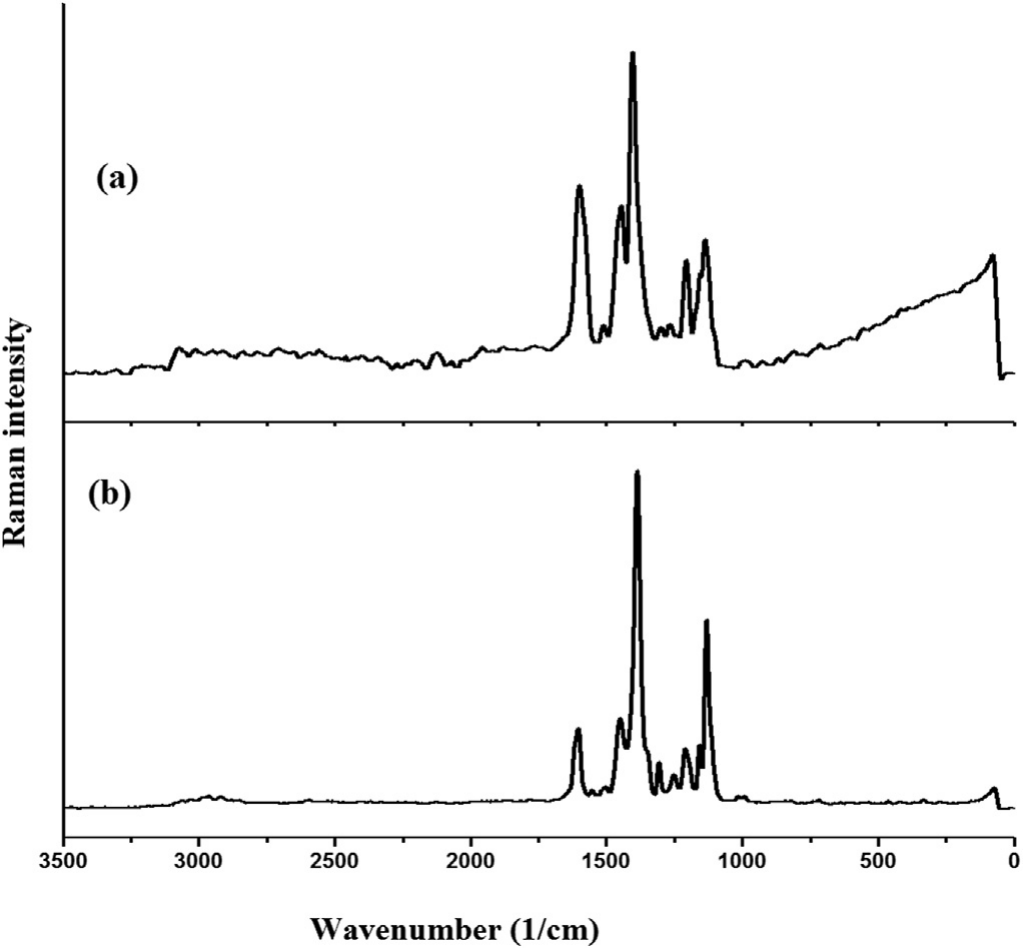
Experimental FT-Raman curves of (a) HMY and (b) HMM.

HMY and HMM are prepared according to reported protocol [21, 22]. Perkin Elmer spectrometer was used for FT-IR spectrum (Figure 2) and Raman spectrum (Figure 3) of the sample were using Bruker UFS 66V model interferometer using Nd:YAG laser source.

## 3 Results and discussion

### 3.1 Conformational studies

**Figure 4:**
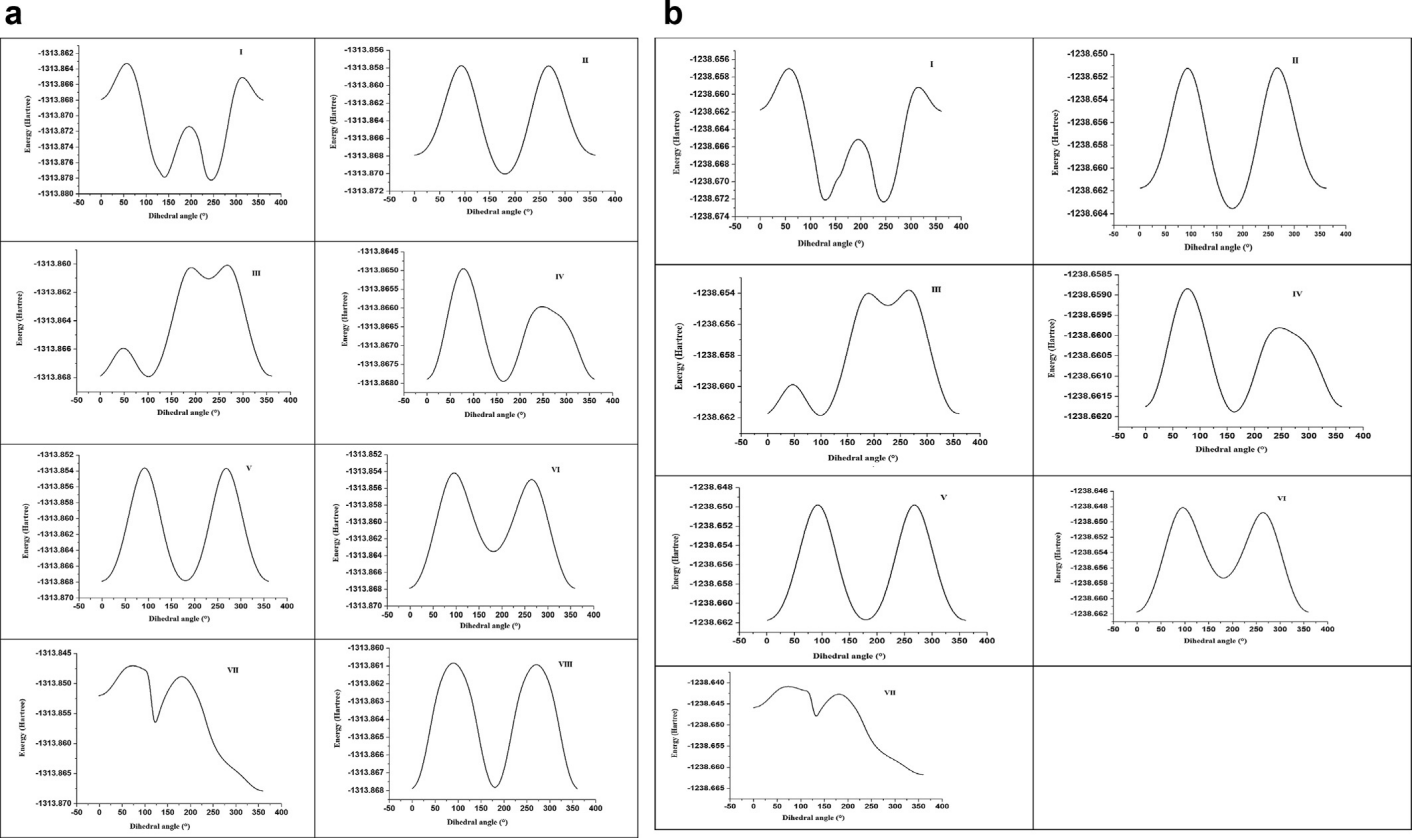
(1): Potential energy surface scan with varying dihedral angle for HMY. (2): Potential energy surface scan with varying dihedral angle for HMM.

**Figure 5:**
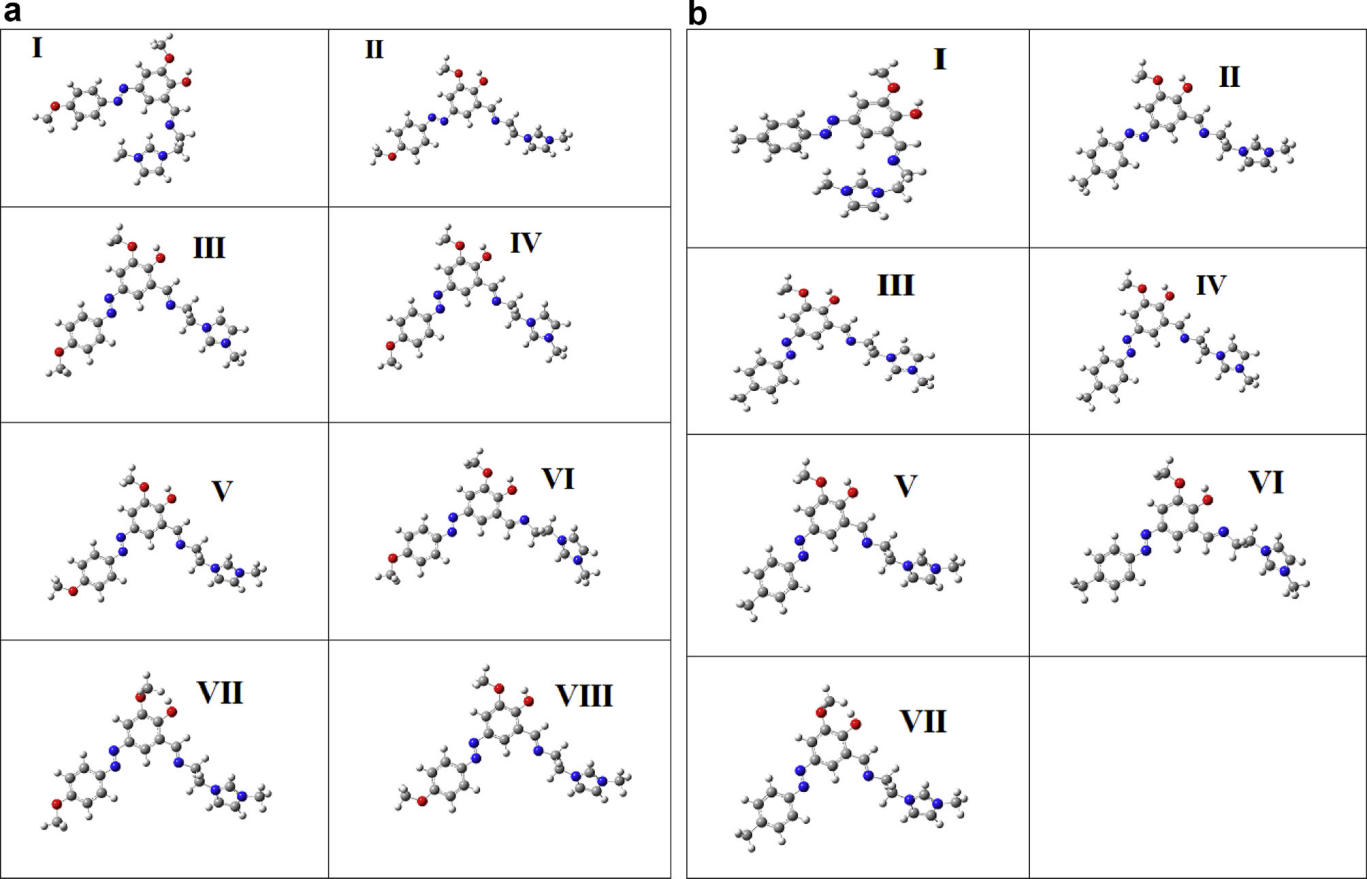
(1): Optimized structure of all the conformers of HMY. (2): Optimized structure of all the conformers of HMM.

To find least energy structure (Figure 1) of HMY and HMM, PES scan is performed at B3LYP/6-311++G (d,p) on C1-N11, N12-C13, C16-C28, N30-C31, C31-C34, C34-N37 and C17-O20. In addition to this C4-O49 is also selected for HMY. The dihedral angles corresponding to these bonds are C6-C1-N11-N12, N11-N12-C13-C14, C14-C16-C28-N30, C28-N30-C31-C34, N30-C31-C34-N37, C31-C34-N37-C38 and C15-C17-O20-C23 for both HMY and HMM with an additional torsion angle C3-C4-O49-C50 for HMY. The structure obtained at global and local minima from the PES graph is again optimized to illustrate most stable conformer. All together 8 and 7 conformers are obtained after optimization corresponding to local and global minima for HMY and HMM. The energy as well as relative energies are predicted (Table 1). The PES curves and energies of structures are shown in Figures 4 and 5. Stable least energy form is along N30-C31-C34-N37 and is calculated to be -1313.87821 and -1238.67223 Hartree for HMY and HMM respectively.

**Table 1:** Ground state optimized energy and energy difference of all the possible conformers of HMY and HMM predicted at B3LYP/6-311++G (d,p) level.

code	Dihedral angle	Conformers	Energy (Hartree)	Energy difference∗ (Hartree)
HMY				
	φ1(N30-C31-C34-N37)	I	-1313.87821	0.00000
	φ2(N11-N12-C13-C14)	II	-1313.87012	0.00809
	φ3(C28-N30-C31-C34)	III	-1313.86797	0.01024
	φ4(C31-C34-N37-C38)	IV	-1313.86796	0.01025
	φ5(C6-C1-N11-N12)	V	-1313.86795	0.01026
	φ6(C14-C16-C28-N30)	VI	-1313.86357	0.01464
	φ7(C15-C17-O20-C23)	VII	-1313.85791	0.0203
	φ8(C3-C4-O49-C50)	VIII	-1313.8679	0.01031

HMM				
	φ1(N30-C31-C34-N37)	I	-1238.67223	0.00000
	φ2(N11-N12-C13-C14)	II	-1238.66361	0.00862
	φ3(C28-N30-C31-C34)	III	-1238.66191	0.01032
	φ4(C31-C34-N37-C38)	IV	-1238.6619	0.01033
	φ5(C6-C1-N11-N12)	V	-1238.66175	0.01048
	φ6(C14-C16-C28-N30)	VI	-1238.65737	0.01486
	φ7(C15-C17-O20-C23)	VII	-1238.64908	0.02315

∗ Relative energies of the other conformers with respect to the lowest energy of conformer I.

### 3.2 AIM analysis

**Figure 6:**
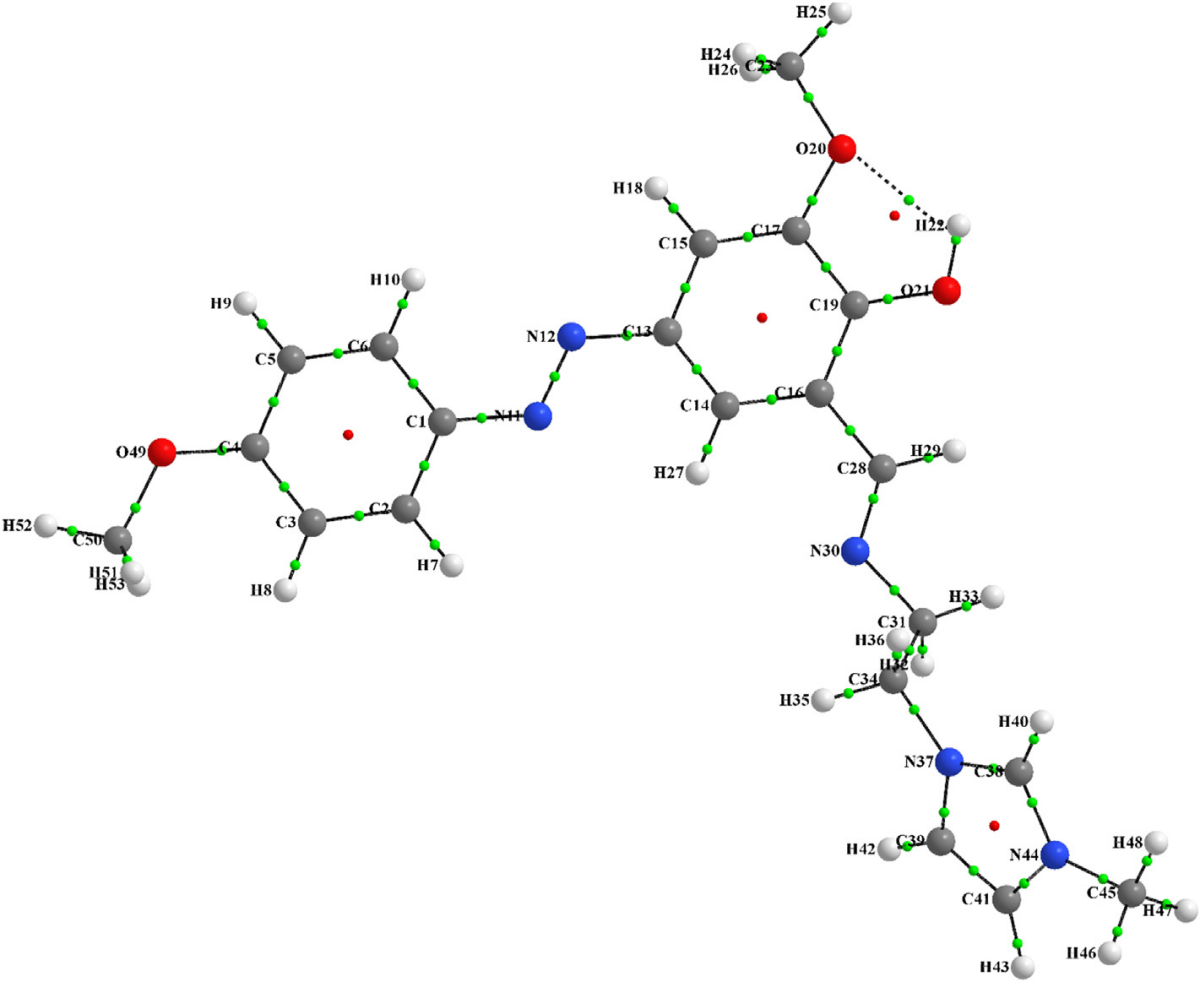
The molecular graph of HMY. Nuclei and bond critical points are represented by big and small spheres small, respectively (green and red circles are bond and ring critical points, respectively). The lines are bond paths.

**Figure 7:**
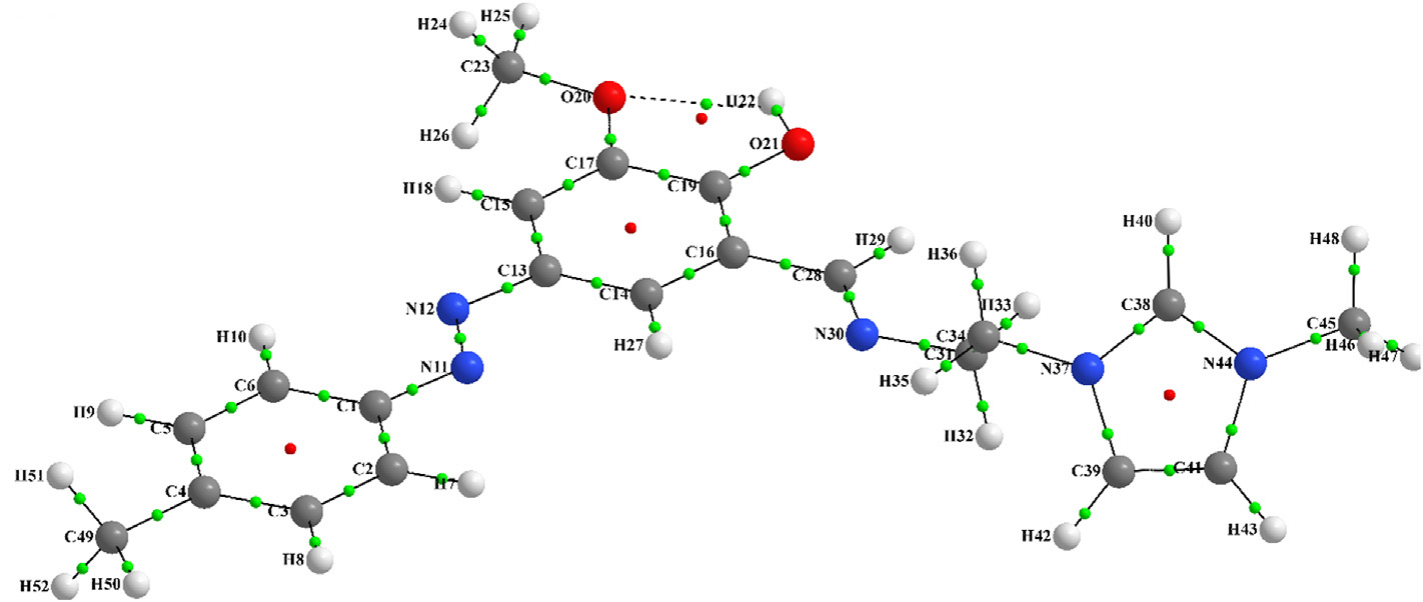
The molecular graph of HMM. Nuclei and bond critical points are represented by big and small spheres small, respectively (green and red circles are bond and ring critical points, respectively). The lines are bond paths.

Atom in molecule (AIM) analysis was used to find inter-molecular interactions. AIM molecular graph of stable geometry of HMY and HMM was illustrated in Figures 6 and 7, respectively, while the status of all BPs and BCPs related to these systems were clearly indicated. The densities (electron and Laplacian) and ellipticity parameters (ε) at critical points for all bonds of HMY and HMM compounds were depicted in Tables 2 and 3, respectively. The values of electron density, 0.022–0.294 au shows a good interaction between atoms. Increase in ρ caused the reduced distance between atoms. The ρ valued for all bonds except O20-H22 in both HMY and HMM are in the range of covalent while for the stated bond is the range of hydrogen bonding. The Laplacian of charge density (∇^2^ρ(r)) for all bonds at critical points also are listed in Tables 2 and 3. There is very good agreement between charge density and Laplacian of charge density. As can be seen in Tables 2 and 3, the Laplacian of charge density for all bonds except O20-H22 is negative which points towards the covalent interaction. For the O20-H22 bond, the value is positive which a proof of noncovalent interaction is. Results of Tables 2 and 3 showed that the ε of O20-H22 had values higher than the ε of the other bonds. This was due to the hydrogen interaction between them.

**Table 2:** The charge density (ρ(r)), Laplacian of charge density (∇^2^ρ(r)), and ellipticities (ε) of all bonds in the HMY molecule.

BCP #	Bonds	ρ(r)	$\nabla^{2}\rho$(r)	ellipticities (ε)
1	C1 - C2	+0.293474	-0.679263	+0.157327
2	C4 - O49	+0.260343	-0.431654	+0.030025
3	C2 - H7	+0.267002	-0.817221	+0.000368
4	C3 - C4	+0.290659	-0.663510	+0.165621
5	O49 - C50	+0.214792	-0.264160	+0.004719
6	C2 - C3	+0.293051	-0.676996	+0.153967
7	C4 - C5	+0.289159	-0.667390	+0.148286
8	C1 - C6	+0.285618	-0.636701	+0.132554
9	C1 - N11	+0.278641	-0.685598	+0.061311
10	C5 - C6	+0.299533	-0.707720	+0.159039
11	C5 - H9	+0.266947	-0.818374	+0.010984
12	C3 - H8	+0.265824	-0.796857	+0.015527
13	C6 - H10	+0.269218	-0.841686	+0.000434
14	N11 - N12	+0.400618	-0.685853	+0.100118
15	N12 - C13	+0.276321	-0.649895	+0.053436
16	C14 - H27	+0.270792	-0.864558	+0.004650
17	C13 - C14	+0.294548	-0.676199	+0.153297
18	C13 - C15	+0.286707	-0.647805	+0.149354
19	C15 - C17	+0.299385	-0.708768	+0.182515
20	C14 - C16	+0.286702	-0.644364	+0.137671
21	C17 - O20	+0.253129	-0.411917	+0.023050
22	C15 - H18	+0.267546	-0.826837	+0.008868
23	C23 - H26	+0.264248	-0.784377	+0.049591
24	C17 - C19	+0.292935	-0.672649	+0.202456
25	O20 - C23	+0.211152	-0.232825	+0.007834
26	C16 - C19	+0.290678	-0.668725	+0.181251
27	C19 - O21	+0.263285	-0.449991	+0.005436
28	C23 - H25	+0.268898	-0.829214	+0.047328
29	O20 - H22	+0.021809	+0.086103	+0.378981
30	O21 - H22	+0.323275	-1.542628	+0.019028
31	C23 - H24	+0.264192	-0.783579	+0.049784
32	C16 - C28	+0.263245	-0.551444	+0.073764
33	C28 - H29	+0.264062	-0.791729	+0.001822
34	C28 - N30	+0.351235	-0.918510	+0.065149
35	N30 - C31	+0.254153	-0.509720	+0.020824
36	C31 - H32	+0.259967	-0.745527	+0.011215
37	C31 - H33	+0.256538	-0.711519	+0.014103
38	C31 - C34	+0.227665	-0.410698	+0.069474
39	C34 - H35	+0.268104	-0.827327	+0.046342
40	C34 - H36	+0.266464	-0.803683	+0.048723
41	N37 - C38	+0.313346	-0.823830	+0.199277
42	C34 - N37	+0.217735	-0.347959	+0.018092
43	N44 - C45	+0.224133	-0.391916	+0.023152
44	C38 - H40	+0.275304	-0.934888	+0.033530
45	C38 - N44	+0.310572	-0.795118	+0.194983
46	N37 - C39	+0.276296	-0.615939	+0.091254
47	C41 - N44	+0.275030	-0.595892	+0.096469
48	C41 - H43	+0.273403	-0.908601	+0.032106
49	C39 - C41	+0.312757	-0.759861	+0.261251
50	C39 - H42	+0.273698	-0.913111	+0.030626
51	C45 - H48	+0.266036	-0.808271	+0.045458
52	C45 - H46	+0.265719	-0.809716	+0.046971
53	C45 - H47	+0.265764	-0.810060	+0.046948
54	C50 - H53	+0.263083	-0.772022	+0.046892
55	C50 - H51	+0.263012	-0.771275	+0.047014
56	C50 - H52	+0.268911	-0.828553	+0.043833

**Table 3:** The charge density (ρ(r)), Laplacian of charge density (∇^2^ρ(r)), and ellipticities (ε) of all bonds in the HMM molecule.

BCP #	Bonds	ρ(r)	$\nabla^{2}\rho$(r)	ellipticities (ε)
1	C4 - C49	+0.237480	-0.450813	+0.016833
2	C1 - C2	+0.293578	-0.680858	+0.152152
3	C2 - H7	+0.266587	-0.812454	+0.000392
4	C3 - C4	+0.290342	-0.660512	+0.144914
5	C49 - H50	+0.258736	-0.733113	+0.012426
6	C2 - C3	+0.294256	-0.684823	+0.144086
7	C4 - C5	+0.286480	-0.642914	+0.132693
8	C1 - C6	+0.287941	-0.648139	+0.134745
9	C1 - N11	+0.275607	-0.669105	+0.048672
10	C5 - C6	+0.296966	-0.694978	+0.148439
11	C5 - H9	+0.264822	-0.791661	+0.005762
12	C3 - H8	+0.264949	-0.791596	+0.007051
13	C49 - H52	+0.255417	-0.713519	+0.013510
14	C6 - H10	+0.268680	-0.834937	+0.000778
15	N11 - N12	+0.402635	-0.694275	+0.099312
16	N12 - C13	+0.275774	-0.647636	+0.052971
17	C14 - H27	+0.270928	-0.866571	+0.004230
18	C13 - C14	+0.294706	-0.677070	+0.152886
19	C13 - C15	+0.286835	-0.648398	+0.149715
20	C14 - C16	+0.286986	-0.645763	+0.137194
21	C15 - C17	+0.299386	-0.708688	+0.182348
22	C15 - H18	+0.267569	-0.827299	+0.008687
23	C17 - C19	+0.292758	-0.671782	+0.201752
24	O20 - C23	+0.211007	-0.231834	+0.007492
25	C16 - C19	+0.290733	-0.669185	+0.180553
26	C17 - O20	+0.253347	-0.412865	+0.023387
27	O20 - H22	+0.021834	+0.086191	+0.379276
28	C19 - O21	+0.263723	-0.451270	+0.006519
29	O21 - H22	+0.323184	-1.542509	+0.018957
30	C23 - H25	+0.268941	-0.829750	+0.047355
31	C23 - H24	+0.264175	-0.783563	+0.049871
32	C23 - H26	+0.264239	-0.784367	+0.049730
33	C16 - C28	+0.263013	-0.550401	+0.073451
34	C28 - H29	+0.264114	-0.792403	+0.001748
35	C28 - N30	+0.351451	-0.916326	+0.065704
36	N30 - C31	+0.254052	-0.509962	+0.020945
37	C31 - H32	+0.259907	-0.745154	+0.011359
38	C31 - H33	+0.256590	-0.712158	+0.014084
39	C31 - C34	+0.227775	-0.411188	+0.069549
40	C34 - N37	+0.217772	-0.348507	+0.018836
41	C34 - H35	+0.268137	-0.827833	+0.046234
42	C34 - H36	+0.266458	-0.803609	+0.048634
43	N37 - C38	+0.313314	-0.823559	+0.199193
44	N44 - C45	+0.224123	-0.391781	+0.023195
45	C38 - N44	+0.310590	-0.795435	+0.195059
46	N37 - C39	+0.276278	-0.615669	+0.091125
47	C41 - N44	+0.275023	-0.595883	+0.096309
48	C38 - H40	+0.275312	-0.934903	+0.033534
49	C41 - H43	+0.273395	-0.908676	+0.032075
50	C39 - C41	+0.312778	-0.759980	+0.261217
51	C39 - H42	+0.273687	-0.913183	+0.030605
52	C45 - H48	+0.266031	-0.808233	+0.045476
53	C45 - H46	+0.265749	-0.810020	+0.046940
54	C45 - H47	+0.265744	-0.809902	+0.046989
55	C49 - H51	+0.255374	-0.713113	+0.013558

### 3.3 Molecular reactivity analyses, electronic spectra, MEP and NLO studies

**Figure 8:**
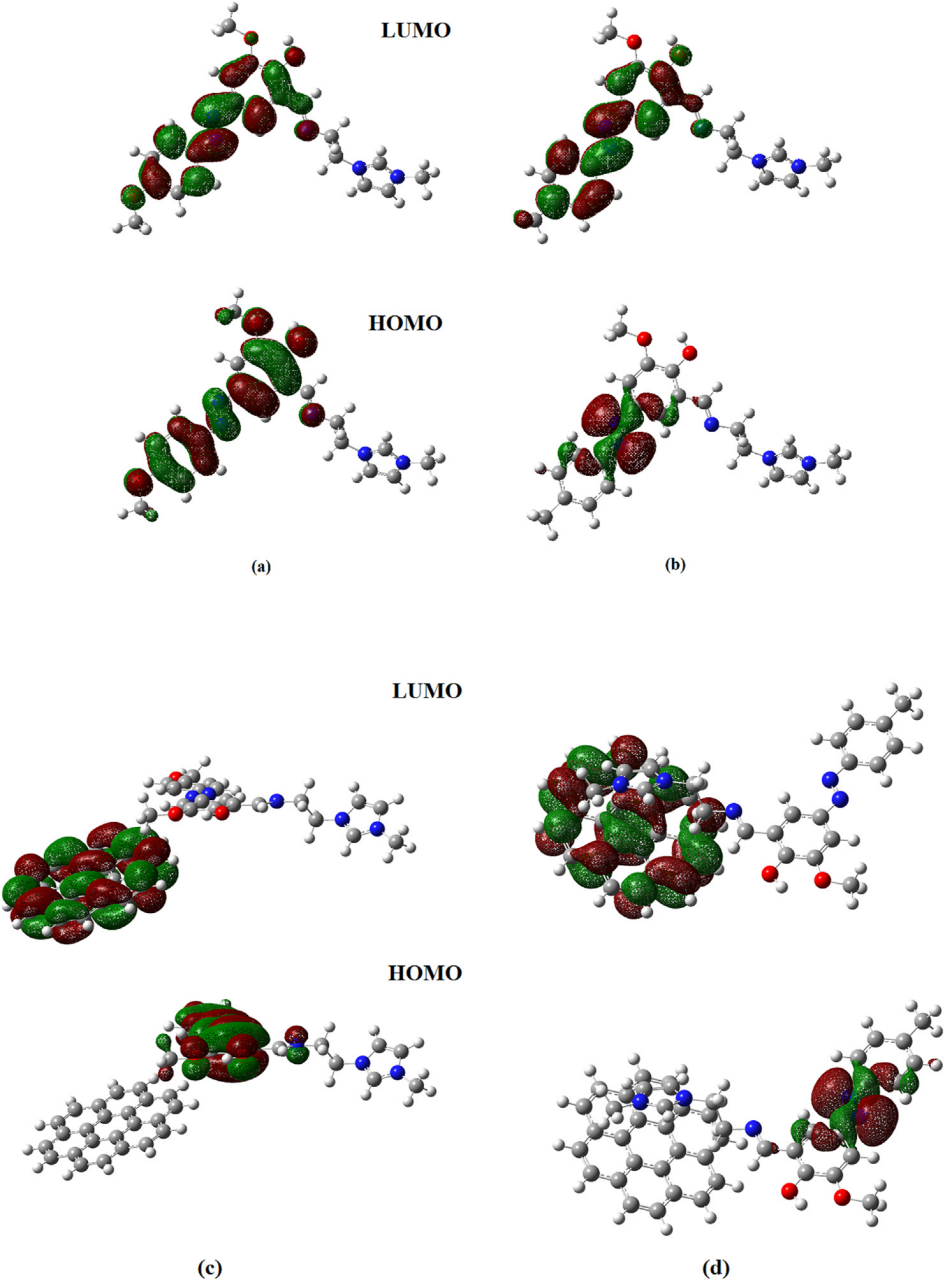
HOMO-LUMO plots of (a) HMY, (b) HMM (c) HMY-G (d) HMM-G.

**Figure 9:**
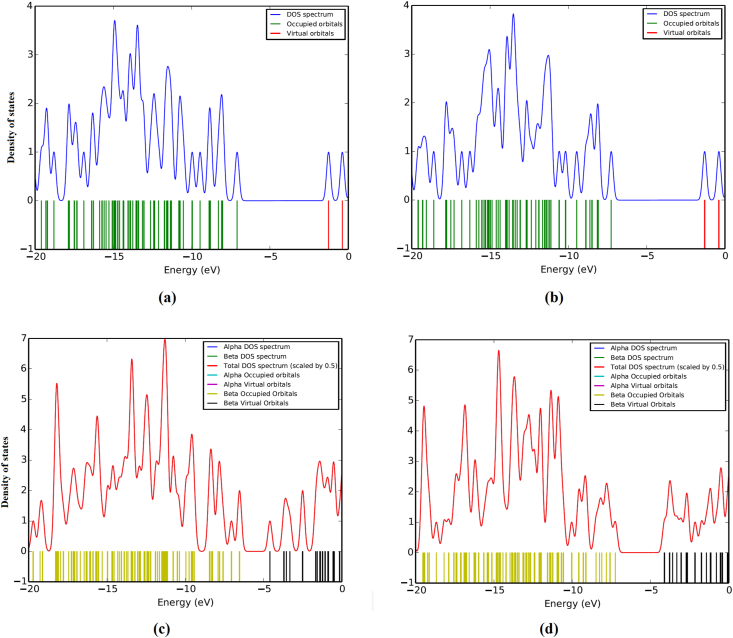
DOS spectra of (a) HMY (b) HMM (c) HMY-G (d) HMM-G.

**Figure 10:**
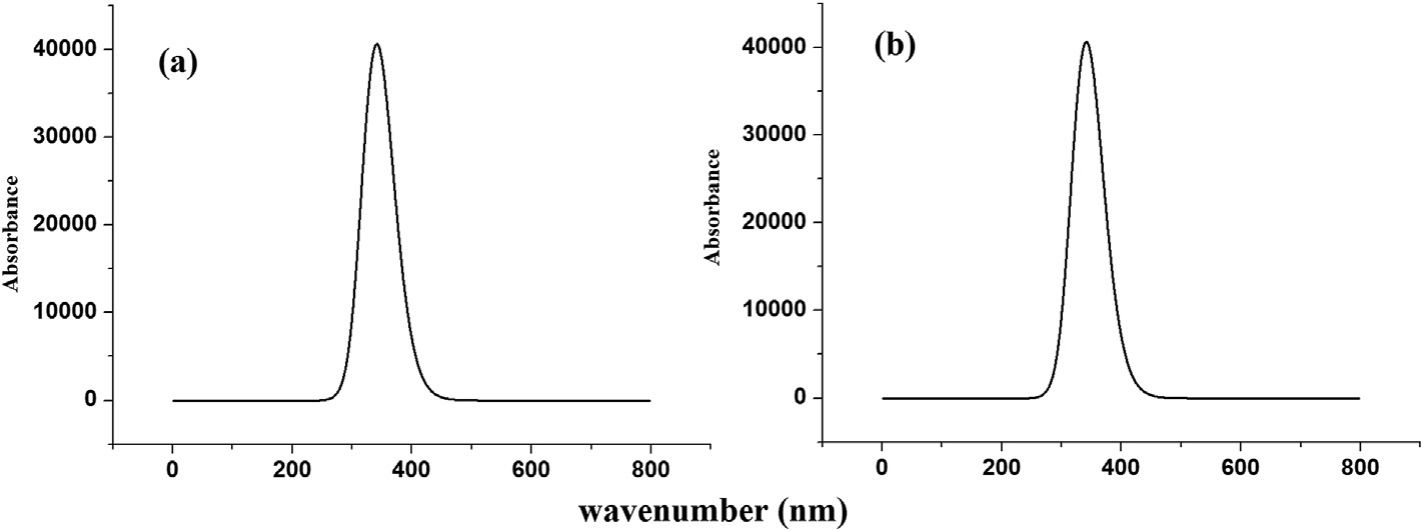
UV spectra of (a) HMY (b) HMM.

**Figure 11:**
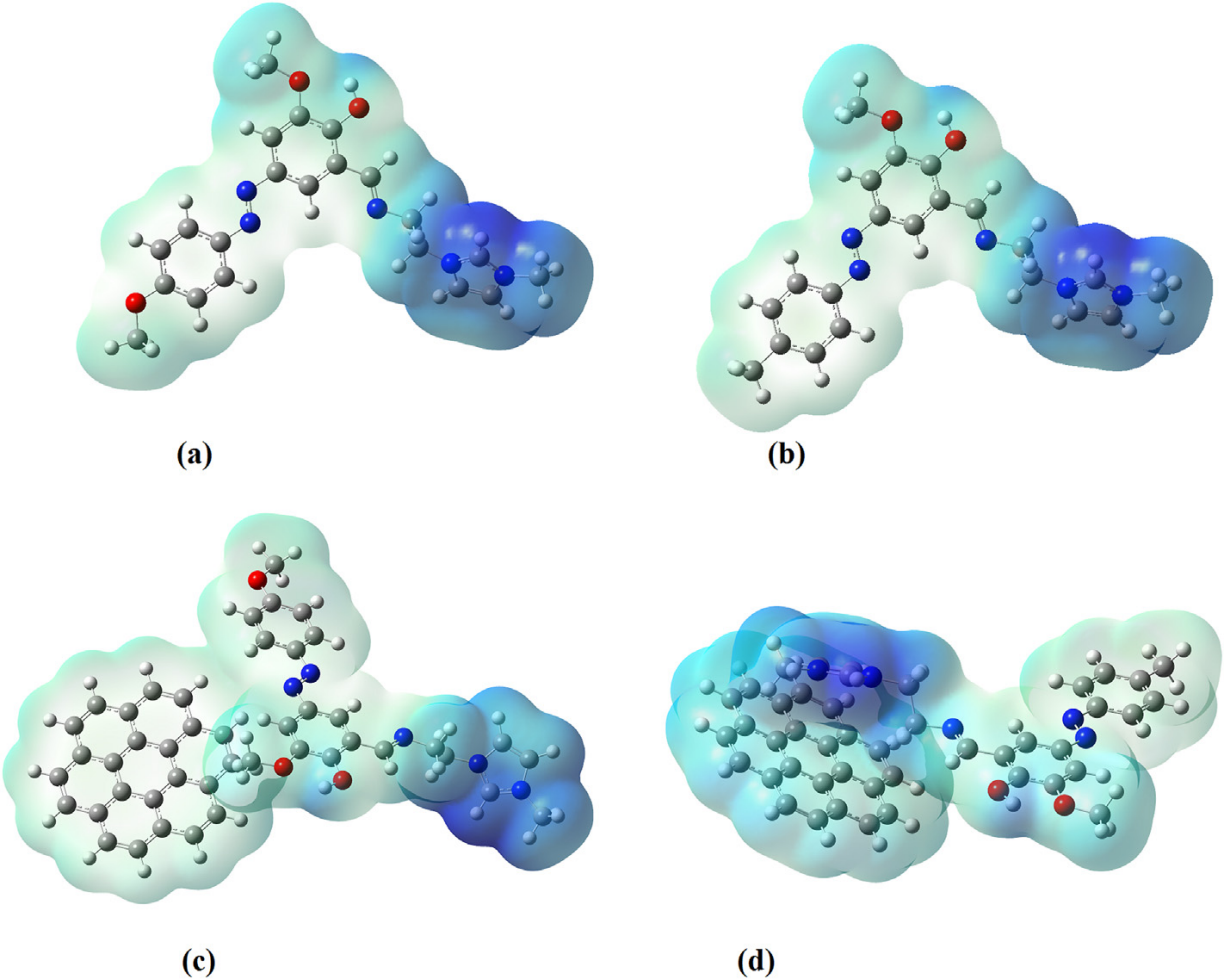
MEP plots of (a) HMY (b) HMM (c) HMY-G (d) HMM-G.

The HOMO-LUMO band gap is a basic parameter in deciding atomic transport effects such as the chemical reactivity of a substance and kinetic stability of a molecule since it is a measure of electron conductivity [73, 74, 75]. Values of band gaps (ΔE_H-L_) of HMY and HMM are 2.381 and 2.240 respectively (Table 4). HOMO (Figure 8), is over entire part of HMY except ring R3, CH2 groups and for HMM, in NN group, C1 and C13 atoms of rings R1 and R2 whereas LUMO spread the same regions as in HOMO of HMY and for HMM except in ring R3, CH2 groups and OCH3 regions. Reactivity descriptors clearly reflect that the title molecules are is chemically reactive [76, 77, 78]. PDOS spectra (Figure 9), shows that no much overlap is present in FMOs but overlap in core orbital. UV-Vis spectra (Figure 10) gives absorption/oscillator strength/LHE values as 350.78, 341.67/1.0927, 0.9950/91.92 and 89.98% for HMY and HMM [79]. The distribution of charge in space around the molecule is predicted from the fitting point charges to the electrostatic potentials [80, 81]. The various colors coding on the MEP surface as shown in Figure 11 indicates the sites feasible for electrophilic and nucleophilic attacks [82]. The yellow color regions around oxygen atoms and rings R1 and R2 in HMY and HMM are electrophilic regions whereas the blue color regions across R3 for both HMY and HMM shows low electron density (nucleophilic). The orientation of charge levels among the molecular site in the compound are usually measured different order such as first and second order [83]. Normally, the first order is measured loosely bounded electron delocalization and they occupied with high pressure generated by interactive and repulsive forces among the molecular sites and leads static chemical potential for regulate drug activity. In the second order, the bounded electrons with strong binding forces with nucleus delocalized with frenkel pressure generates enforced asymmetrical polarization called hyperpolarization causing intra atomic static potential which is the root cause of sensitive drug commotion. First order hyperpolarizability (10^−30^ esu) of HMY (40.147) is greater than that of HMM (24.359) which are 308.82 and 187.38 times that of urea while next order values are -30.166 × 10^−37^ esu and -28.116 × 10^−37^ esu for HMY and HMM (Table 5). Here the first order polarization for both chain and ring was enabled and causing strong drug hardness capability. Here, the second order polarizability of the compound was also enables strongly which showed stabilization of consistent drug potential in the compound and this compound was able to have additional ligand groups for making multifunctional drug movement [84].

**Table 4:** Chemical descriptors.

Compound	I = -EHOMO	A = -ELUMO	Gap	η=(I-A)/2	μ = -(I + A)/2	ω = μ^2^/2η
HMY	7.822	5.441	2.381	1.191	-6.632	18.465
HMM	7.833	5.593	2.240	1.120	-6.713	20.118
HMY-G	7.813	5.732	2.081	1.041	-6.773	22.033
HMM-G	7.887	5.738	2.149	1.075	-6.813	21.589

**Table 5:** NLO properties.

Molecule	Dipole moment	Polarizability	First order	Second order
-	(Debye)	-	hyperpolarizability	hyperpolarizability
		(×10^−23^)	(×10^−30^)	(×10^−37^)
HMY	25.787	4.785	40.147	-30.166
HMM	23.843	4.589	24.359	-28.116
HMY-G	40.784	8.952	45.493	-61.096
HMM-G	12.352	8.435	19.521	-88.326

### 3.4 NBO analysis

The important NBO [85] interactions are: O49→π∗(C3-C14), N44→π∗(C39-C41), N44→π∗(N37-C38), O21→π∗(C16-C19), O20→π∗(C15-C17) with energies, 31.98, 29.08, 78.82, 29.30, 26.27 kcal/mol for HMY and N44→π∗(N37-C38), N44→π∗(C39-C41), O21→π∗(C16-C19), O20→π∗(C15-C17), C16-C19→π∗(C28-N30), C16-C19→π∗(C13-C14), C15-C17→π∗(C16-C19) with energies, 78.86, 29.07, 29.48, 26.34, 20.94, 20.41, 20.42 kcal/mol for HMM.

### 3.5 Molecular docking

**Figure 12:**
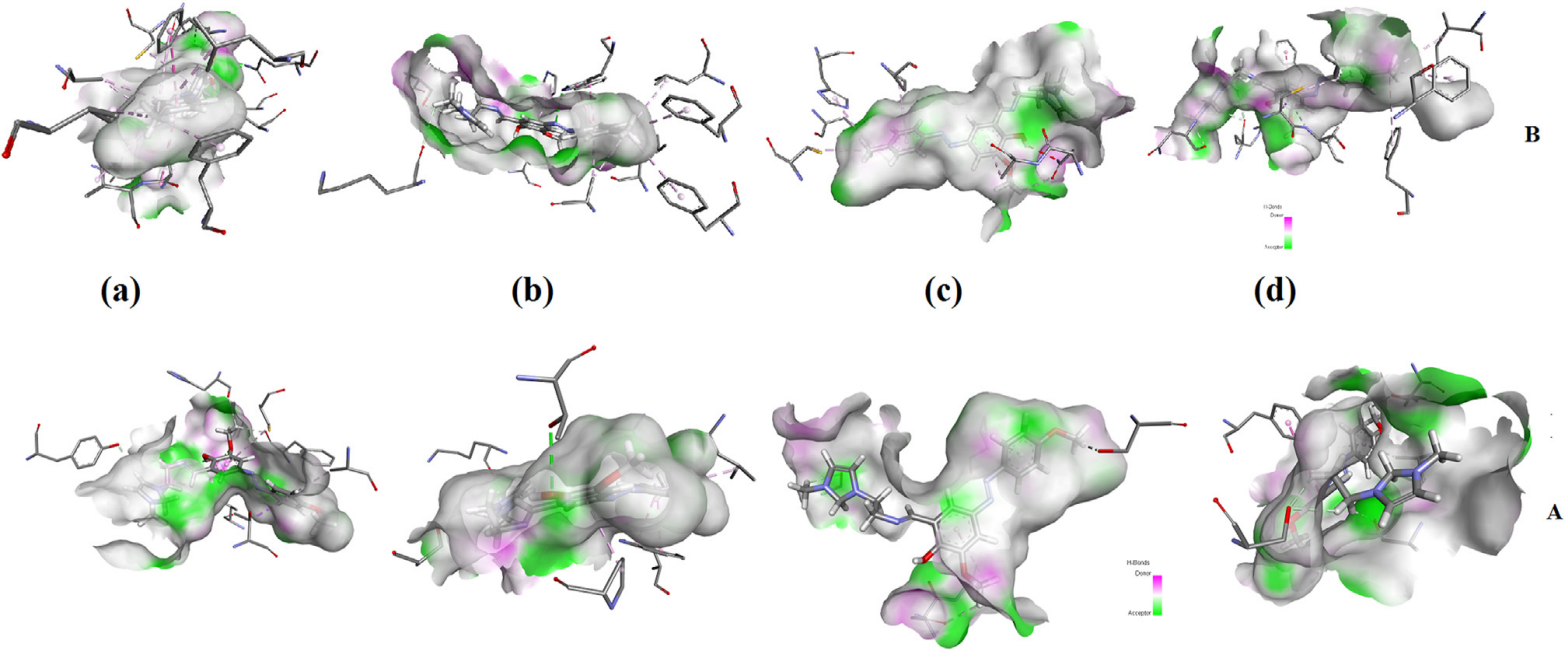
The docked ligands HMY (A) and HMM (B) interaction with the amino acids of (a) 1QMQ (b) 4JB4 (c) 2WYA (d) 4KF0.

**Figure 13:**
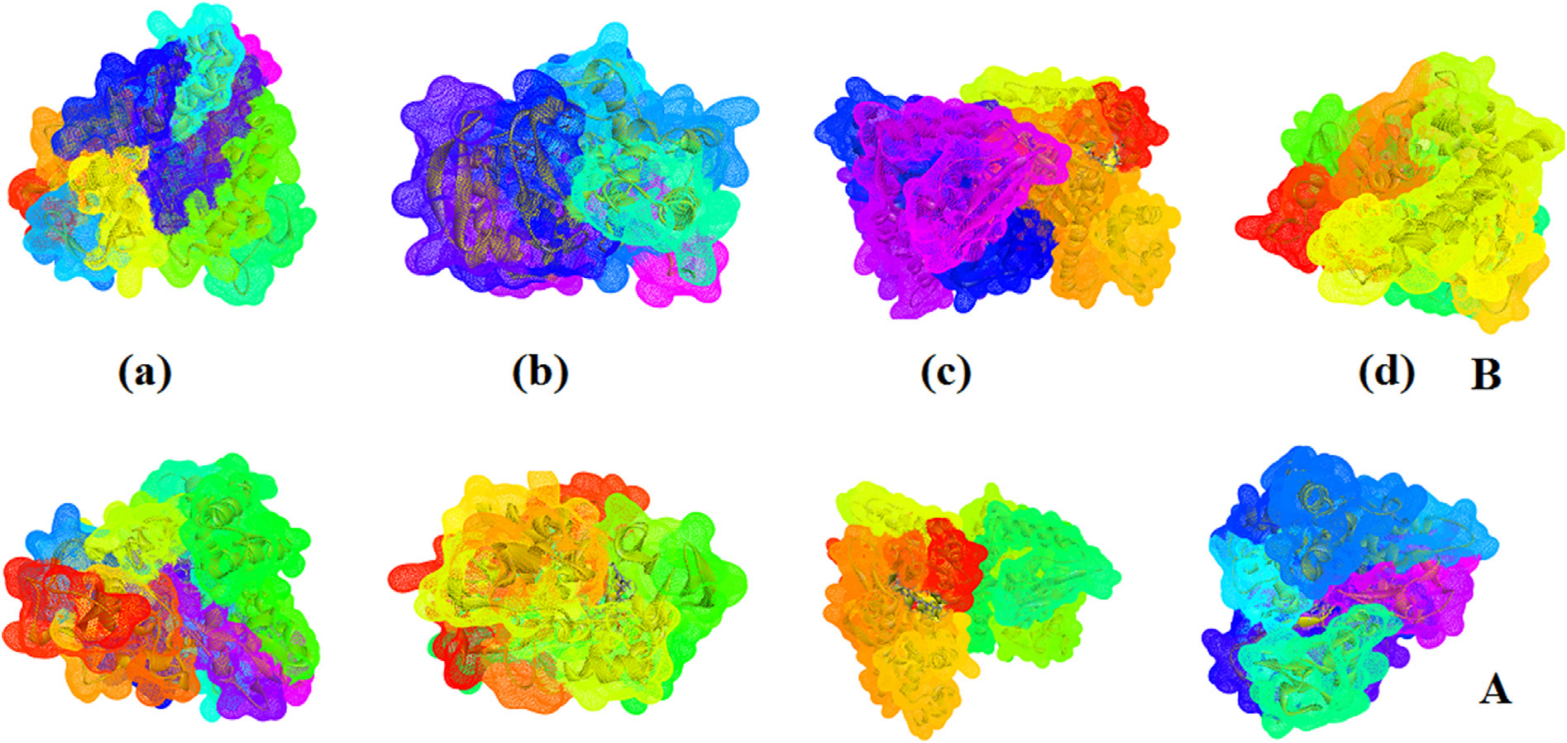
Schematic for the ligands HMY (A) and HMM (B) at the active site of a) 1QMQ (b) 4JB4 (c) 2WYA (d) 4KF0.

To explore the drug properties, ligand-protein interaction is essential to be investigated to get more insights into the binding sites of biologically active molecules with amino acids of the protein and docking has been carried out by using Autodock software [86]. Targets, Chemo sensitizer, APOA1 expression enhancer, HMGCS2 expression enhancer and CYP2C19 inducer as predicted by online PASS analysis [87] for HMY and HMM. Using the PDB's, 1QMQ, 4JB4, 2WYA, 4KF0, HMY and HMM are docked [88, 89]. Lamarckian Genetic Algorithm [90] included in Autodock software has been implemented for docking. Interactions are presented in Figures 12 and 13. The maximum energy values of HMY and HMM are -9.8 and -10.7 kcal/mol for 4JB4 respectively (Table 6). These results suggest that HMY and HMM shows activity against these cytochrome C peroxidase. However further experimental studies are needed to confirm its activity.

**Table 6:** Docking analysis of receptors with ligands.

Molecule	Receptors name	Binding energy (kcal/mol)	Residues involved in hydrogen bonding	Residues involved in electrostatic interactions	Residues involved in hydrophobic interactions
HMY	1QMQ	-8.9	ASP297, GLN322, TYR96, PHE350, HIS355	ASP297	THR252, VAL295, CYS357, ALA363
	4JB4	-9.8	THR234, SER185, LYS179	TRP51,	TRP51, PRO145, LEU171, ALA174
	2WYA	-8.1	SER414, GLY87, ASN204		ALA205, PRO303
	4KF0	-8.3	THR268, SER72, ALA330	SER72, CYS400	PHE87, ALA328
HMM	1QMQ	-9.5	ASP297, GLN32, VA; 247, PHE350, HIS355,	GLN322, ASP297, ALA363, PH350	VAL295, PHE350, VAL253, LEU289, ALA363, ILE 367, CYS357, PHE256,
	4JB4	-10.7	THR234, TRP51, SER185, LYS179, LEU171, ALA174	TRP51	TRP51, LEU171, ALA174, LEU269, PRO145, PHE262, PHE266
	2WYA	-8.1	ASN204, TYR412		CYS166, ALA205, PRO303,HIS301
	4KF0	-9.0	LYS69, ARG398, ASN395, ILE401,	ARG398, CYS400	CYS400, PHE87, ILE153, ALA264, PHE107, PHE405

### 3.6 IR and Raman spectra

A sharp peak observed at 3435, 3430 cm^−1^ and 3447 cm^−1^ experimentally (Table S1) and at 3451 cm^−1^ (DFT) are υOH [91, 92]. δOH was 1250 (DFT) and seen at 1251/1252 cm^−1^ (IR) for HMY/HMM and at 1248 cm^−1^ (Raman) for HMM. τOH is at 544 cm^−1^ (Raman) for HMM but DFT give this at 539 (HMY) and 542 cm^−1^ (HMM). The prominent peaks observed at 3068, 3002, 2960, 2925, 2850/3205, 3067, 3011, 2958, 2918, 2850 cm^−1^ and 3180, 3128, 3020, 2865, 2934, 2850/3130, 2970, 2922, 2850 cm^−1^ (IR/Raman) are υCH modes and DFT values are 3221-2850/3220-2858 cm^−1^ for HMY and HMM respectively. The υC = CR3 is assigned at 1547 cm^−1^ and at 1549/1547 cm^−1^ (IR) spectrum for HMY and HMM.

### 3.7 Adsorption behavior on graphene sheet

**Figure 14:**
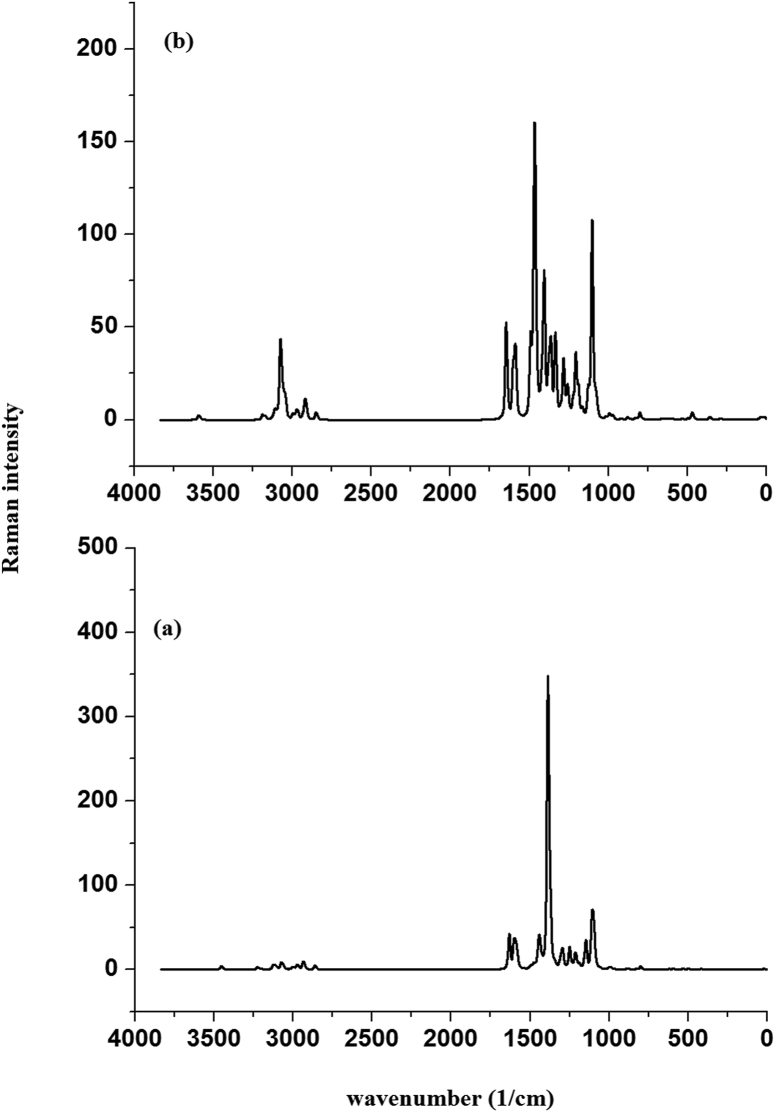
Theoretical Raman spectra of (a) HMY (b) HMY-G.

**Figure 15:**
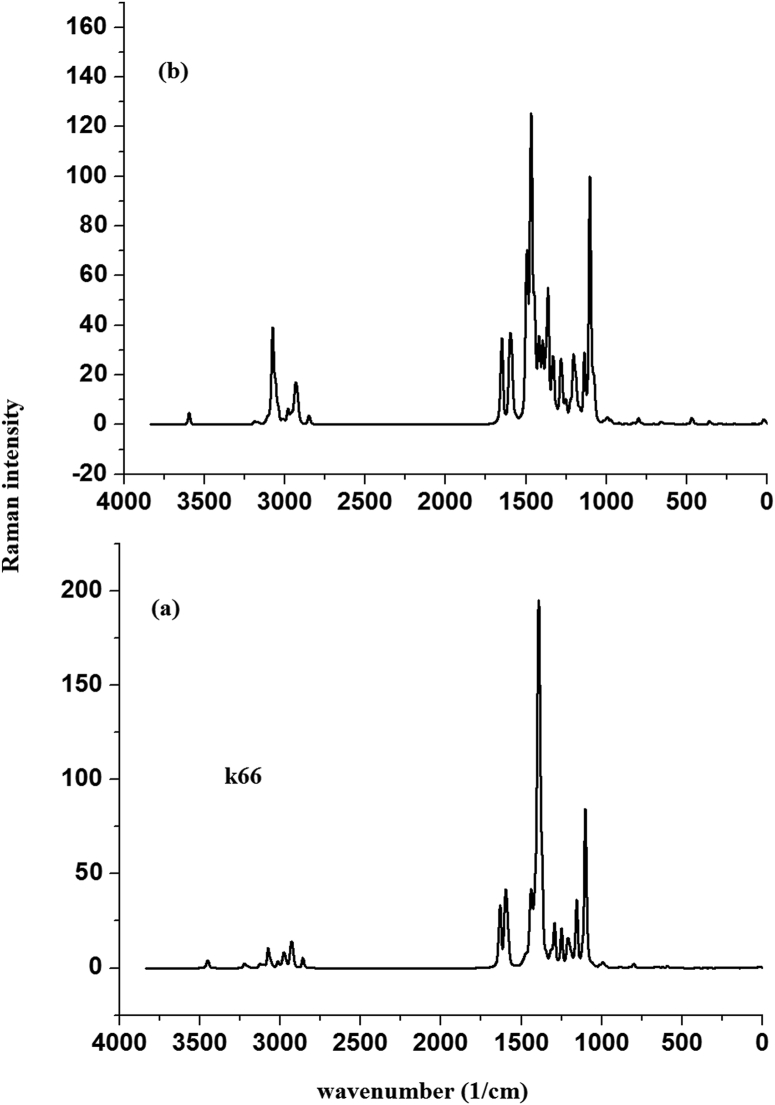
Theoretical Raman spectra of (a) HMM (b) HMM-G.

Adsorption process of graphene-drugs is reported by many researchers [93, 94, 95, 96, 97, 98]. Adsorption energies are -0.65833 and -0.63937 eV for HMY-G and HMM-G complexes [99]. Chemical potential and electrophilicity indices (Table 4) for graphene complex with HMY and HMM shows more stability. The HOMO-LUMO plots and MEP plots (Figures 8 and 11) show the charge transfer between graphene sheet and molecules with a lowering of hardness values. Table 5 data shows that NLO property of graphene-MHY/HMM increase and hyperpolarizability values are very high. For HMY/HMM-G complexes (Table S2), intensity multiplication is for 3074 cm^−1^ from 10.05/12.92 to 46.04/40.55, with enhancement factors, 358/214, which is significant (Figures 14 and 15). Multiplication of 237 and 230 are seen for 1440 cm^−1^ and 1348 in HMY-G complex, corresponding to CH3 and CH2 deformations with blue and redshift to 1463 and 1336 cm^−1^. But for HMM-G, enhancement of these deformation modes is high and enhancement factors of 1172 and 1168 for the modes 1486 cm^−1^ and 1468 cm^−1^. For both complexes υCH3, υC = N and υRing modes are in SERS spectrum with low enhancement factors. Enhancement of different modes shows that graphene can be used as sensor for detection of drugs [100, 101].

## 4 Conclusion

In the present work, quantum chemical descriptors, and vibrational spectral studies of HMY and HMM were studied. Conformational analysis identifies the most stable conformer. The theoretically obtained data are in agreement with experimental results. The FMO's determined energy gap shows the chemical stablility. NBO results account for the natural charge accumulation in the investigated molecule. QTAIM study shows that ellipticity of OH bond had values higher than the ellipticity of the other bonds which is due to hydrogen interactions. SERS data of HMY/HMM with graphene gives enhancement of Raman signals.

Docking simulation obtained shows good binding affinities with the receptors. The predicted docked models will be the starting point of drug discovery. This study could hopefully serve as a base for further drug research and development.

## Declarations

### Author contribution statement

Veena S. Kumar, Y. Sheena Mary, Kiran Pradhan, Dhiraj Brahman: Contributed reagents, materials, analysis tools or data; Wrote the paper.

Y. Shyma Mary, Goncagül Serdaroğlu, Ali Shokuhi Rad, M. S. Roxy.

### Funding statement

This research did not receive any specific grant from funding agencies in the public, commercial, or not-for-profit sectors.

### Competing interest statement

The authors declare no conflict of interest.

### Additional information

No additional information is available for this paper.

## Appendix A Supplementary data

TableS1=S2.docx TableS1=S2.docx
