# Supplementary material for: Conformational analysis and quantum descriptors of two new imidazole derivatives by experimental, DFT, AIM, molecular docking studies and adsorption activity on graphene
Source: Heliyon. 2020 Oct 6;6(10):e05182. doi: 10.1016/j.heliyon.2020.e05182 (PMC7548447; doi:10.1016/j.heliyon.2020.e05182)
Supplement: TableS1=S2.docx [file mmc1.docx]

Conformational analysis and quantum descriptors of two new imidazole derivatives by experimental, DFT, AIM, Molecular docking studies and adsorption activity on graphene

Veena S.Kumar^a^, Y.Sheena Mary^b*^, Kiran Pradhan^c^, Dhiraj Brahman^c^, Y.Shyma Mary^b^,

Goncagül SERDAROĞLU^d^, Ali Shokuhi Rad^e^, M.S.Roxy^a^

^a^ Department of Physics, SN College, Kollam, Research Centre, University of Kerala, Kerala, India

^b^ Department of Physics, Fatima Mata National College(Autonomous), Kollam, Kerala, India

^c^Department of Chemistry, St. Joseph's College, P.O. North Point, Dist. Darjeeling-734104, India

^d^Sivas Cumhuriyet University, Faculty of Education, Math.and Sci. Edu., 58140 Sivas/ TURKEY

^e^Department of Chemical Engineering, Qaemshahr Branch, Islamic Azad University, Qaemshahr, Iran

Author for correspondence: [marysheena2018@rediffmail.com](mailto:marysheena2018@rediffmail.com)

Table S1

Table S1.1 Vibrational Assignments of HMY

B3LYP/6-31G (6D, 7F) IR Raman Assignments^a^

υ(cm^-1^) IR_I_ R_A_ υ(cm^-1^) υ(cm^-1^) -

3451 202.0 246.8 3435 3447 υOH(100)

3221 5.32 9.18 - - υCHR3(99)

3210 3.89 14.94 - - υCHR3(91)

3201 13.62 51.10 - 3205 υCHR3(99)

3139 2.16 5.33 - - υCHR2(99)

3126 5.46 9.63 - - υCHR1(97)

3125 2.61 4.64 - - υCHR2(98)

3119 16.13 1.23 - - υCHR1(98)

3108 2.60 1.10 - - υCHR1(98)

3098 6.05 4.55 - - υCHR1(98)

3074 0.74 5.35 - - υCH3(100)

3072 14.78 1.95 - - υCH3(100)

3069 17.70 206.2 3068 3067 υCH3(90)

3059 0.88 8.57 - - υCH3(99)

3051 2.14 2.06 - - υCH2(99)

3010 2.68 44.21 - 3011 υCH3(99)

2998 36.01 7.09 3002 - υCH3(99)

2992 5.55 4.57 - - υCH2(99)

2973 5.45 1.38 - - υCH3(100)

2962 31.92 69.21 2960 2958 υCH(99)

2935 20.34 2.91 - - υCH2(99)

2934 3.32 1.83 - - υCH3(100)

2925 63.83 165.9 2925 2918 υCH3(100)

2857 96.29 199.0 2850 2850 υCH2(99)

1632 280.9 1276.7 1627 1650 υC=N(62), δCH(16)

1601 269.7 977.7 1602 1600 υR1(63), δCHR1I(22)

1597 3.37 1.60 - - υR2(63), δCHR2(14)

1584 148.7 8.19 1583 - υR2(49), δCHR2(17)

1560 8.34 2.06 - - υR1(63), δCHR1(16)

1547 18.39 6.66 1549 - υC=C(45), υCNR3(13), δCHR3(19)

1533 1.78 7.52 - - υCNR3(44), δCHR3(15), δCH2(12)

1496 57.05 88.74 1500 1507 δCHR1(14), υR1(44)

1487 16.29 2.38 - - δCH3(88)

1485 9.00 12.49 - - δCH3(76)

1483 23.65 12.93 - - δCH2(88)

1480 7.59 9.30 - - δCH3(89)

1475 11.31 4.47 - - δCH3(95)

1472 28.39 34.24 - - δCH3(89)

1471 1.51 1.06 - - δCH3(32), υR2(34)

1470 23.19 26.88 - - δCH2(79)

1468 19.20 23.87 1468 - δCH3(94)

1449 3.03 442.4 - 1449 δCH3(47), υR2(20)

1440 23.77 2.61 1441 - δCH3(73), δCHR1(10)

1437 12.46 6.44 - - δCH3(41), υNN(11), υR2(12)

1435 17.31 2.27 - - δCH3(74)

1424 16.74 1.13 - - υR1(31), δCHR1(27), υR2(12)

1392 26.55 1053.75 - 1400 δCH(34), υNN(13), δCH2(24)

1387 1.47 8.72 - - υNN(51), δCH(10)

1380 1.88 6.83 - - υCNR3(59), δCH2(17)

1374 4.55 21.66 - - υR2(56), δOH(19)

1364 2.61 5.60 - - υCNR3(38), δCH2(22)

1348 21.69 1.29 - - δCH2(47), υCC(12)

1341 47.64 5.58 1342 - υR1(78)

1315 2.68 17.41 - - δCH2(55), υCNR3(10)

1306 11.66 3.49 - - δCHR1(77)

1295 2.00 605.6 - 1296 υR2(38), υCC(10), υCO(14)

1287 7.20 2.71 - - δCH2(31), υCN(46)

1279 1.45 19.32 1277 - δCHR3(52), δCH2(22)

1264 1.52 50.95 - 1266 δCH2(57), δCHR3(27)

1250 153.2 5.10 1251 - δOH(33), δCHR2(28)

1236 5.57 105.1 - 1240 υCO(49), υR1(15), δCH3(14)

1228 5.57 4.73 - - υCC(34), δCHR2(37)

1212 46.58 681.7 1210 1209 υCN(35), δCHR1(32)

1187 43.94 1.41 1185 - δOH(22), δCHR2(19), υCO(38)

1169 7.36 9.25 - - δCH2(68)

1161 2.15 9.16 - - δCH3(72)

1158 25.23 6.31 - - δCH3(64), δCHR1(10)

1148 275.2 910.7 1147 1150 δCHR1(54), υCN(13)

1136 1.14 6.48 - 1136 δCHR3(28), υCN(36), υCNR3(13)

1132 0.59 0.87 - - δCH3(94)

1123 0.49 7.52 1125 - δCH3(96)

1118 0.38 8.23 - - δCH3(96)

1111 4.49 9.74 - - δCHR1(41), υCN(12), υR1(11)

1105 13.80 6.36 - - δCHR3(67), υCC(18)

1099 10.41 2.15 1100 - υCN(38), δCHR2(15), δCHR1(11)

1081 17.09 4.55 - - δCH2(54), υCNR3(10)

1075 1.87 12.05 - - δCH3(55), υCNR3(16)

1058 15.73 3.72 - - υCO(50), υCC(14)

1040 3.40 9.51 - 1040 υCC(37), δCH2(48)

1011 6.65 7.24 1016 - δR3(20), υCNR3(43)

1003 16.74 24.38 - - δR3(29), υR2(15), υCN(16)

1000 3.43 10.61 - - υCNR3(38), δR1(13), δR3(12)

999 4.44 6.09 - - δR1(33), υR1(21)

993 0.37 1.44 - - γCHR1(84), τR1(10)

990 8.58 43.44 - - γCH(28), υCC(25)

978 10.88 2.54 - 980 υCO(74)

975 5.76 24.09 975 - υCN(56), υCC(13)

957 0.57 2.83 - - γCHR1(82), τR1(10)

944 8.55 4.61 - 930 γCHR2(76), τR2(10)

897 47.70 3.97 - - υCO(44), δR2(13)

895 31.34 1.42 - - γCHR2(74), τR2(11)

880 1.34 33.34 - - υR1(29), δCN(17), δNN(14)

879 0.03 3.21 - 873 γCHR3(83), τR3(16)

852 56.26 1.63 855 - γCHR1(86), τR1(10)

826 33.41 10.18 827 - γCHR3(72), τR3(23)

820 8.76 4.11 - - γCHR1(95)

801 15.77 71.60 - 807 υCO(17), δR2(35)

793 2.18 12.91 - - δCH2(74)

773 20.19 2/58 774 773 δR1(38), υCO(18)

755 24.38 1.41 - - γCHR3(80), γCN(19)

734 27.90 2.98 - - τR2(26), δR3(10), δCH2(13)

727 5.14 0.80 - - τR1(61), γCO(14), γCN(13)

718 3.34 3.66 - 717 τR2(46), γCO(40)

706 1.06 3.89 - - δR2(31), δR1(21)

663 28.37 8.24 - 661 τR3(25), δCO(10), δR2(22)

651 12.11 2.16 - - τR3(41), γCN(19)

639 6.20 10.16 - - δR1(74), δCN(10)

630 11.84 1.50 - - γCN(46), τR2(30)

627 16.55 0.33 625 - τR3(75), γCN(20)

618 8.29 9.26 - - τR3(28), δR3(42)

593 5.04 17.29 - - δR2(29), δR1(23)

563 0.37 0.42 561 557 γCO(33), τR2(32), γCC(21)

539 15.73 3.99 - - τOH(94)

531 73.44 14.80 530 - δR2(33), δCO(29)

525 10.15 0.08 - - τR1(28), γCO(28), γCN(23)

497 5.55 17.62 - 500 δR1(13), δCO(13), δCN(16), δR2(24)

493 23.35 5.48 - - δR2(25), δCO(35), δCN(12)

436 0.14 0.13 - 441 τR1(69), τR2(12)

418 12.53 10.02 - 418 δR1(18), δCO(29), δR2(12)

415 0.50 1.09 - - δCN(59)

412 1.13 0.80 - - γCO(37), τR1(27)

403 9.17 1.58 - - δCH2(28), δCN(20)

395 11.62 1.19 - - δCN(21), δCH2(29)

381 2.73 3.46 - 383 τR1(31), γCO(24), γCN(14)

342 6.81 2.00 - - δCO(43), δR2(17)

322 3.05 2.58 - 322 δCO(29), τCN(29)

297 7.25 2.04 - - δCO(29), δCN(27)

284 1.67 1.08 - - τR2(29), τCH3(32), γCO(14)

273 3.72 3.47 - - δCN(57)

268 3.54 3.80 - - γCN(24), τR2(21)

238 1.54 0.31 - - τCH3(53), τR1(24)

231 14.32 5.62 - 229 γCN(22), δCH2(19), τCC(20)

213 10.16 2.00 - - δCO(63)

203 0.93 2.57 - - τCH3(51), τR2(15)

201 1.26 5.92 - - γCN(24), τCH3(21)

189 3.86 0.84 - - τR1(40), τCH3(26)

178 2.34 0.78 - - τCH3(21), δR1(28)

172 2.22 1.11 - 169 τR2(33), τCH3(14), τCC(18)

149 1.63 6.48 - - δCO(37), δCN(33)

127 5.07 1.92 - - τCC(30), τCO(25)

119 5.71 2.04 - - τCO(39), τCH3(15)

112 4.53 1.18 - - τCO(42), τCN(15), τCH3(18)

88 2.03 1.52 - - γCN(19), τCH2(51)

76 0.33 0.51 - 75 τCO(29), τR1(12), τCH3(23)

66 1.75 0.67 - - τCO(29), τCH3(28), τCN(14)

60 0.29 0.74 - - τCH3(57), γCN(16)

53 0.91 0.67 - - τCH2(35), τCC(20)

38 1.09 0.77 - - τCH2(32), γCN(16)

35 0.80 0.83 - - τNN(42), γCN(16)

27 2.92 1.68 - - τCN(27), τCH2(30)

22 0.13 7.86 - - τCN(59), τCH2(10)

19 1.13 3.13 - - τCH2(55), τCN(10)

14 2.35 5.09 - - τCH2(39), τCN(11), τR1(21)

^a^υ-stretching; δ-in-plane deformation; γ-out-of-plane deformation; τ-torsion; IR_I_-IR intensity(KM/Mole) ; R_A_-Raman activity(Ǻ^4^/amu);R1 and R2-para and poly phenyl rings; R3-imidazole ring.

Table S1.2 Vibrational Assignments of HMM

B3LYP/6-31G (6D, 7F) IR Raman Assignments^a^

υ(cm^-1^) IR_I_ R_A_ υ(cm^-1^) υ(cm^-1^) -

3451 198.54 2.74 3430 - υOH(100)

3220 4.81 1.13 - - υCHR3(98)

3210 32.71 14.06 - - υCHR3(90)

3200 13.64 49.93 3180 - υCHR3(99)

3140 2.47 5.96 - - υCHR2(99)

3126 2.42 54.02 3128 3130 υCHR2(99)

3119 3.64 4.26 - - υCHR1(96)

3104 11.16 7.63 - - υCHR1(99)

3077 22.90 12.75 - - υCHR1(98)

3074 0.75 5.38 - - υCH3(99)

3073 14.36 1.90 - - υCH3(100)

3072 20.73 14.05 - - υCHR1(95)

3059 0.87 8.25 - - υCH3(100)

3051 2.14 28.45 - - υCH3(98)

3014 18.95 9.01 3020 - υCH3(99)

3010 27.52 4.49 - - υCH3(100)

2992 5.39 4.89 - - υCH2(99)

2980 16.32 1.61 - - υCH3(100)

2973 5.41 187.04 - 2970 υCH3(99)

2964 30.85 63.77 2965 - υCH(99)

2935 14.73 2.72 - - υCH2(95)

2934 40.55 9.62 2934 - υCH3(97)

2924 30.04 580.8 - 2922 υCH3(99)

2858 93.98 193.10 2850 2850 υCH2(100)

1633 274.9 11.04 1640 - υC=N(62), δCH(16)

1600 7.56 1168.60 - 1602 υR1(58), δCHR1(17)

1597 4.79 2.42 - - υR2(62), δCHR2(14)

1584 15.15 6.66 - - υR2(49), δCHR2(17)

1565 7.95 0.79 - 1560 υR1(68), δCHR1(14)

1547 19.13 6.51 1547 - υC=C(44), υCNR3(14), δCHR3(19)

1533 8.62 6.84 - - υCNR3(44), δCHR3(25)

1496 14.31 27.46 - 1506 δCHR1(14), υR1(43)

1487 16.31 2.62 - - δCH3(89)

1486 7.80 11.02 - - δCH3(78)

1483 23.09 13.85 - - δCH2(90)

1478 15.33 33.26 - - δCH3(84)

1472 15.55 34.75 - - δCH3(93)

1471 19.52 12.81 - - δCH3(28), υR2(28), υCO(10)

1471 9.22 34.99 - - δCH3(99)

1470 15.60 28.72 - - δCH2(83)

1468 19.57 22.27 - - δCH3(96)

1449 5.70 311.25 1450 1450 δCH3(53), υR2(19)

1437 31.25 11.72 - - δCH3(27), υNN(16), υR2(10)

1435 17.06 22.18 - - δCH3(74)

1417 30.19 9.41 - - υR1(32), υNN(10), δCHR1(19)

1406 3.15 3.34 - - δCH3(89)

1394 18.49 4328.23 - 1392 υNN(43), υR1(13), δCH(12)

1389 74.27 2174.54 1387 1392 δCH(35), υNN(12), δCH2(16)

1380 2.02 5.02 - - υCNR3(54), δCH2(17)

1374 36.59 15.39 - - υR2(52), δOH(19)

1364 2.91 5.94 - - υCNR3(39), δCH2(17)

1347 21.65 10.42 - - δCH2(53), υCNR3(11)

1327 3.27 21.88 - - υR1(86)

1315 1.91 209.80 - 1316 δCHR1(80)

1314 22.65 22.05 - - δCH2(55), υCN(14)

1295 24.95 6.16 - - υR2(32), υCC(18)

1286 74.46 19.48 - - δCH2(32), υCN(44)

1279 1.26 15.10 - - δCHR3(54), δCH2(20)

1263 1.93 48.27 - - δCH2(58), δCHR3(26)

1250 227.1 450.75 1252 1248 δOH(33), δCHR2(28)

1229 17.81 19.74 - - υCO(44), δCHR2(43)

1212 23.30 323.84 1215 1214 υCN(33), δCHR1(37)

1203 23.00 31.44 - - υCC(39), υR1(36)

1187 38.33 12.21 - - δOH(22), δCHR2(20), υCO(39)

1169 8.52 11.38 - - δCH2(61)

1163 38.44 78.16 1165 - δCH3(60)

1156 86.01 884.21 - 1158 υCN(37), δCHR1(32), δCH3(13)

1137 14.85 6.95 - - δCHR3(28), υCN(36), υCNR3(13)

1131 0.03 1.04 - 1132 δCH3(93)

1124 0.48 6.96 - - δCH3(95)

1118 26.99 18.14 1120 - δCHR1(61), υR1(20)

1104 17.21 28.01 - - δCHR3(66), υCC(17)

1101 29.67 22.88 - - υCN(33), δCHR2(19)

1081 14.14 29.61 - - δCH2(54)

1074 1.63 11.26 - - δCH3(55), υCNR3(26)

1058 144.33 39.39 - - υCO(48), υCN(17)

1056 8.84 0.25 - - δCH3(64), δCHR1(29)

## 1039 2.95 9.28 - - υCC(38), γCH(24), δCH2(23)

1015 18.66 13.30 1020 1020 δR1(25), δR3(17), υR1(11)

1011 5.46 12.79 - - δR1(20), δR3(25)

1003 17.23 24.58 - - υR2(23), δR2(20)

1000 3.39 8.27 - - υCNR3(39), δR3(23)

993 4.93 10.88 - 995 δCH3(51), υR1(10)

991 1.91 2.78 - - γCHR1(83), τR1(10)

989 9.80 40.91 - - γCH(30), υCC(23)

975 8.51 20.08 - - υCO(56), υCC(21)

966 1.06 2.71 965 - γCHR1(84), τR1(10)

946 8.13 4.36 - - γCHR2(83)

896 43.31 4.18 - - υCO(50), δR2(10)

895 29.43 1.45 - - γCHR2(75), τR2(10)

882 2.99 15.18 - - υR1(17), δCN(27), δNN(19)

879 0.03 2.99 - - γCHR3(82), τR3(11)

847 6.41 4.96 - - γCHR1(89)

836 27.18 1.04 838 - γCHR1(69), τR1(16)

827 33.49 9.26 - 828 γCHR3(62), τR3(23)

802 17.89 62.81 - - δR2(40), υCO(25)

794 3.50 6.77 - - δCH2(72)

780 7.64 1.89 - - υCC(15), δR1(13), υR1(18), δCH2(12)

755 24.91 1.43 753 - γCHR3(100)

734 21.73 2.26 - - τR2(12), δR3(22), δCH2(19)

721 7.04 0.14 - - τR2(30), τR1(36), γCO(18)

718 1.33 4.21 - 716 τR1(31), τR2(24), γCO(19), γCN(11)

708 4.25 4.23 - - δC=N(18), δR1(43)

665 26.92 17.91 664 - τR3(10), δCO(15), δR2(20)

652 11.95 3.43 - - τR3(43), γCN(14)

643 2.65 9.42 - 645 δR1(70), τR3(10)

630 11.93 1.11 - - γCN(40), τR2(41)

627 17.44 0.30 625 - τR3(75), γCN(20)

618 8.82 8.53 - - τR3(29), δR2(27)

591 17.88 21.11 - - δR2(26), δCO(15), δCN(14)

563 0.32 0.59 562 - γCO(34), τR2(32), γCC(20)

542 156.75 3.98 - 544 τOH(94)

523 24.93 5.99 - - δR2(37), δCO(18), τR2(12)

516 9.61 0.24 - - τR1(30), γCN(27), γCC(19)

500 2.67 1.84 - - δCN(37), δCO(11), δR1(18)

465 26.15 0.20 462 463 δR1(33), δR2(31)

420 0.79 0.38 - - τR1(76)

415 0.43 1.06 - - δCN(55), τR1(12)

410 2.60 1.40 - 409 γCO(42), τR2(16)

401 14.58 1.08 - - δCH2(19), δCN(18), τR2(25)

396 10.25 0.60 - - δCN(31), δCH2(15), δR2(16)

361 2.19 3.58 - - γCC(21), τR1(34), δNN(11)

354 0.61 1.46 - - δCC(33), δCO(14), δR2(15)

327 13.78 2.46 - 330 δCC(34), δCO(36)

320 1.07 2.37 - - τR2(21), τCN(10), δCN(14)

283 1.64 1.25 - 282 τCH3(17), τR2(24), γCO(14), τCN(13)

283 0.37 2.95 - 282 γCN(22), τR2(15), δCN(15)

269 2.93 1.35 - - δCN(44), τR2(10)

256 0.46 4.01 - - γCN(18), δCO(14), δR2(17)

229 14.89 6.08 - - δCH2(16), γCN(16), τCC(16)

206 0.41 0.61 - - τCH3(56), τCN(21)

201 2.51 6.82 - - γCN(26), τR2(32)

191 0.08 0.47 - - τR1(35), γCC(18), τCH3(16)

183 3.72 0.84 - - τNN(13), τR1(31)

173 1.22 1.23 - - τR2(48), τCH3(19)

158 3.95 3.78 - - δCO(40), δCN(28)

127 9.79 2.37 - - τCC(23), τCN(20), δCC(12), γCN(11)

114 1.65 1.12 - - τCO(40), τCC(10), τCH3(13)

91 0.37 0.96 - - τR1(21), τCN(25), τR2(18)

89 1.94 1.69 - 85 γCN(18), τCH2(21), τCO(19)

67 2.02 0.93 - - τCO(37), τCN(21), τCH3(21)

58 0.13 0.82 - - τCH3(51), γCN(14)

52 0.24 0.43 - - τCH3(36), γCN(15), τCH2(18)

41 0.25 0.26 - - τNN(33), γCN(29), τR1(11)

38 1.82 1.42 - - τCH2(37), τCN(10), γCN(19)

27 1.84 1.20 - - τCN(37), τCH3(15)

23 0.53 6.27 - - τCH3(48), γCC(16)

17 1.00 4.27 - - τCH3(49), γCC(18)

15 1.26 4.51 - - τCH2(58)

13 1.91 4.13 - - τCH2(21), τCN(20), τCH3(22)

^a^υ-stretching; δ-in-plane deformation; γ-out-of-plane deformation; τ-torsion; IR_I_-IR intensity(KM/Mole) ; R_A_-Raman activity(Ǻ^4^/amu);R1 and R2-para and poly phenyl rings; R3-imidazole ring.

Table S2.1 Enhancement factor for HMY

| HMY | | HMY-G | | Assignments | Enhancement  Factor |
| --- | --- | --- | --- | --- | --- |
| υ(cm^-1^) | R_I_ | υ(cm^-1^) | R_I_ |  |  |
| 3074 | 10.05 | 3079 | 46.04 | υCH3 | 358 |
| 2925 | 10.86 | 2915 | 13.29 | υCH3 | 22 |
| 1632 | 46.89 | 1644 | 54.67 | υC=N | 17 |
| 1597 | 40.53 | 1582 | 41.24 | υR2 | 2 |
| 1440 | 47.86 | 1463 | 161.41 | δCH3 | 237 |
| 1348 | 14.43 | 1336 | 47.66 | δCH2 | 230 |
| 1295 | 24.82 | 1290 | 33.28 | υR2 | 34 |
| 1212 | 22.71 | 1212 | 37.11 | υCN | 63 |
| 1105 | 77.56 | 1105 | 109.48 | δCHR3 | 41 |

Table S2.2 Enhancement factor for HMM

| HMM | | HMM-G | | Assignments | Enhancement  Factor |
| --- | --- | --- | --- | --- | --- |
| υ(cm^-1^) | R_I_ | υ(cm^-1^) | R_I_ |  |  |
| 3074 | 12.92 | 3079 | 40.55 | υCH3 | 214 |
| 2924 | 17.42 | 2936 | 18.74 | υCH3 | 8 |
| 1633 | 33.50 | 1652 | 36.79 | υC=N | 10 |
| 1486 | 5.54 | 1496 | 70.49 | δCH3 | 1172 |
| 1468 | 10.04 | 1466 | 127.28 | δCH3 | 1168 |
| 1315 | 10.04 | 1332 | 28.72 | δCHR1 | 186 |
| 1295 | 23.54 | 1286 | 27.99 | υR2 | 19 |
| 1212 | 18.89 | 1208 | 28.72 | δCHR1 | 52 |
| 1104 | 86.17 | 1110 | 100.74 | δCHR3 | 17 |
